# Modeling High-Risk Pediatric Cancers in Zebrafish to Inform Precision Therapy

**DOI:** 10.1158/2767-9764.CRC-25-0080

**Published:** 2025-07-25

**Authors:** Nadine Azzam, Jamie I. Fletcher, Nicole Melong, Loretta M.S. Lau, Emmy M. Dolman, Jie Mao, Gabor Tax, Roxanne Cadiz, Lissandra Tuzi, Alvin Kamili, Biljana Dumevska, Jinhan Xie, Jennifer A. Chan, Donna L. Senger, Stephanie A. Grover, David Malkin, Michelle Haber, Jason N. Berman

**Affiliations:** 1 Children’s Hospital of Eastern Ontario Research Institute, Ottawa, Canada.; 2 Children’s Cancer Institute, Lowy Cancer Research Centre, UNSW Sydney, Sydney, Australia.; 3 School of Clinical Medicine, UNSW Sydney, Sydney, Australia.; 4 Kids Cancer Centre, Sydney Children’s Hospital, Randwick, Australia.; 5 Arnie Charbonneau Cancer Institute, Calgary, Canada.; 6 Department of Oncology, McGill University, Montreal, Canada.; 7 Lady Davis Institute for Medical Research, Montreal, Canada.; 8 The Hospital for Sick Children, Toronto, Canada.; 9 Department of Pediatric, University of Toronto, Toronto, Canada.; 10 Department of Medical Biophysics, University of Toronto, Toronto, Canada.; 11 Department of Pediatrics, University of Ottawa, Ottawa, Canada.; 12 Department of Cellular and Molecular Medicine, University of Ottawa, Ottawa, Canada.

## Abstract

Despite advances in precision medicine, 30% of high-risk pediatric cancers lack an actionable molecular target, hindering effective treatment and affecting survival outcomes. Although mouse patient-derived xenograft (PDX) models offer additional insights into clinical drug responses, delivering findings from these models within a clinically actionable time frame remains challenging. This international collaboration between two national precision medicine programs demonstrates proof-of-principle that individualized larval zebrafish PDXs can robustly and rapidly assess clinical responses in high-risk pediatric cancers. Retrospective zebrafish PDX testing was performed on tumor samples from 10 pediatric patients with high-risk cancers. Drug responses in zebrafish models were correlated with clinical responses for each patient and directly compared with responses in cognate mouse PDX models. Responses to conventional and targeted therapies, administered as single agents or in combinations, were assessed. Zebrafish PDXs were successfully established from all 10 patients and provided robust drug response data in every case, including from three patients whose tumor samples could not be engrafted in mice. Remarkably, zebrafish models accurately recapitulated patient responses for 11 of 12 treatment regimens. These findings highlight the potential of larval zebrafish PDX models to provide real-time, clinically relevant drug response data, supporting their potential use in prospective precision medicine studies.

**Significance:**

This proof-of-principle study is the first to compare drug responses in larval zebrafish and mouse PDX models with patient outcomes in pediatric precision oncology, showing high concordance. Results highlight the potential of zebrafish PDX models to predict drug responses in high-risk cancers more accurately, rapidly, and cost-effectively in prospective studies.

## Introduction

Improving treatment outcomes for young patients with high-risk cancers is of paramount importance. With personalized medicine, tailored therapeutic strategies can be developed to address the specific molecular alterations and biological characteristics of an individual patient’s cancer, leading to improved treatment outcomes with the goal of higher survival rates for these children (1–4). However, although precision-guided treatment recommendations based on comprehensive molecular profiling can significantly improve clinical outcomes (5), approximately 30% of high-risk patients with cancer lack actionable molecular targets and hence cannot receive personalized treatment guidance based on comprehensive molecular profiling alone (1, 5). Individualized preclinical models can be effectively leveraged as an avenue for therapeutic validation of putative driver lesions identified by molecular profiling and can also serve as a patient-specific agnostic functional drug screening platform when no candidate aberrations have been identified (6). In both scenarios, personalized treatment approaches can be prioritized or refined.

Patient-derived xenograft (PDX) models in immune-deficient mice are a widely used preclinical tool in cancer research with a valuable role in precision oncology. Individualized mouse PDX models largely preserve the genetic identity of the patient’s tumor, allow robust modeling of drug response using an approach that parallels clinical evaluation, and can enable the study of clonal dynamics and tumor evolution (7). However, delivery of individualized data to actively inform clinical management remains challenging. Mouse PDX models are resource-intensive to develop and maintain, cannot be readily established for all patients, and often have very long latencies (8, 9) that are not consistent with real-time delivery of personalized treatment recommendations. For pediatric solid tumors, these challenges are often increased by the need to expand solid tumor xenografts from a limited needle core biopsy. When a mouse PDX model can be successfully established, there are uncertainties around relevance of preclinical results generated for a patient whose tumor may have evolved under the selective pressure of additional therapies while the model was being established, often over a period of months.

Conversely, although high-throughput drug screens conducted using dissociated tumor cells can rapidly and robustly identify drug sensitivities, clinicians may be hesitant to recommend treatment based solely on *in vitro* data (10). There is therefore a pressing need for validated, orthogonal methods for individualized drug sensitivity testing that are reliable, clinically relevant, and possess the advantages of mouse PDX models while being rapid and cost-effective. Such methods should provide personalized treatment recommendations in real-time and should first be validated through rigorous side-by-side comparisons with mouse PDX testing, correlated with clinical response data. Additionally, platforms offering personalized recommendations for combination therapies, in addition to single agents, are crucial, as novel combination treatment regimens present the best opportunity for sustained response (11, 12).

In recent years, the larval zebrafish has emerged as a robust and efficient model for human tumor xenotransplantation and personalized medicine. Zebrafish share remarkable genetic similarity with humans, possessing approximately 70% homology to human genes (13, 14). They offer several advantages as a low-cost experimental model, including high fecundity, rapid organogenesis, and external development, also making them an attractive platform for high-throughput drug screening with clear potential for cancer treatment optimization (15). Zebrafish larvae are optically transparent and lack an adaptive immune system until 28 days post-fertilization (dpf), making them an attractive animal model with no requirement for immunosuppression. Recently, several proof-of-concept studies have successfully utilized the larval zebrafish in many xenotransplantation studies modeling solid (16, 17) and liquid tumors (18, 19). The larval zebrafish platform has also proven to be valuable for drug discovery (20, 21) and has the potential to personalize cancer therapy (22–24). However, a comprehensive comparison of responses to either conventional or molecularly targeted therapies in zebrafish and mouse PDX models with corresponding patient response data has not been previously undertaken, particularly in the context of pediatric cancers.

**Figure 1:**
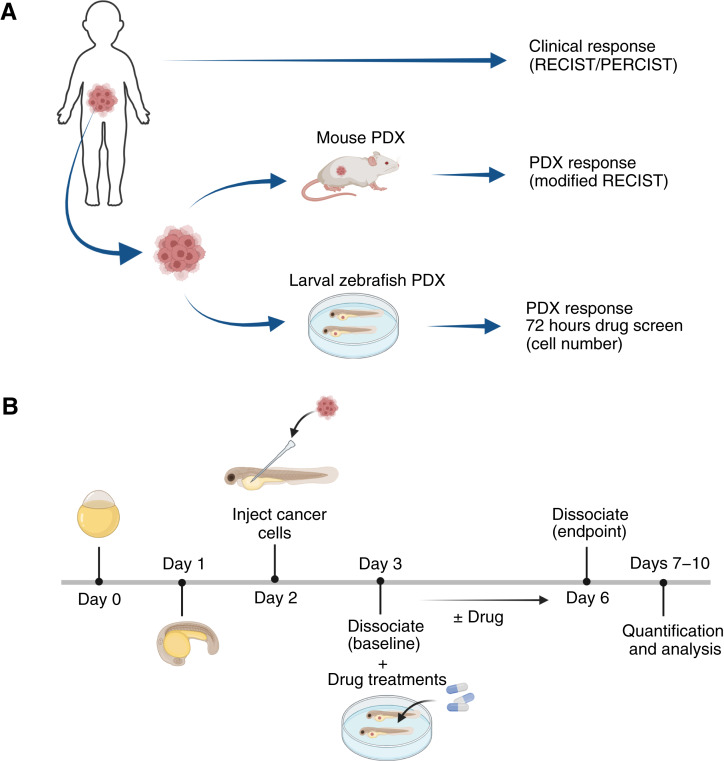
Overview of study design. **A,** Clinical responses in individual pediatric patients with cancer (RECIST/PERCIST criteria) were compared with drug responses in each patient’s cognate preclinical models–mouse PDX (RECIST criteria) and larval zebrafish PDX (viable cell number count). Primary tissue was obtained via biopsy or surgery as part of routine clinical care and frozen for storage or used directly for implantation. Tumor tissues were subcutaneously embedded in the mouse for expansion and drug testing. Cryopreserved tumor tissue or single cells from patient or mouse PDX were shipped from Australia to Canada for larval zebrafish xenograft generation. Injected larval zebrafish were treated with drug by immersion therapy. **B,** Larval zebrafish were injected with dissociated single tumor cells at 2 days post-fertilization (dpf), and immersion therapy was performed on 3 dpf injected fish for a 3-day treatment. Dissociation and quantification of tumor cells were performed at 3 dpf (baseline) and 6 dpf (after treatment). [Created in BioRender. Berman, J. (2025) https://BioRender.com/nbc71w1.]

Zero Childhood Cancer (ZERO) in Australia and PRecision Oncology For Young peopLE (PROFYLE) in Canada are national pediatric cancer precision medicine programs, focused on real-time patient recruitment and generation of personalized treatment recommendations, with the aim of improving outcomes of children with high-risk cancers. Here, in an international collaboration between ZERO and PROFYLE, we conducted the first proof-of-principle study directly comparing single-agent and combination treatment responses of tumor cells from children enrolled on ZERO, using individualized PDX models in larval zebrafish and in mice, and correlating these responses with those observed in the cognate patients to single-agent and combination personalized treatment recommendations (Fig. 1A). Our results from 10 pediatric patients with diverse tumor types show that the drug responses in larval zebrafish PDX models, which were successfully established from every patient, were highly correlated with patient response and performed comparably to mouse PDX models. Our findings suggest that the larval zebrafish PDX model has the potential to provide rapid, cost-effective, and clinically actionable data in real-time that can inform treatment decisions and enhance personalized medicine for pediatric patients with cancer in the future. By demonstrating the process and the concordance of the zebrafish PDX models with both mouse PDX results and clinical responses in patients, this study establishes a foundation for their prospective use.

## Materials and Methods

### Patient samples

The PRISM clinical trial (NCT03336931), conducted as part of the Australian ZERO Precision Medicine Program, was approved by the Hunter New England Human Research Ethics Committee of the Hunter New England Local Health District (reference no. 17/02/015/4.06) and the New South Wales Human Research Ethics Committee (reference no. HREC/17/HNE/29). Informed consent for each participant was provided by parents/legal guardian for participants under the age of 18 years and by the participants who were over the age of 18 years. Tumor tissue was engrafted into mice either directly or following initial cryopreservation. Criteria for inclusion in this study were based on evaluable clinical response—complete response (CR), partial response (PR), or stable disease (SD)—or a lack of response [progressive disease (PD)] to a single-agent or combination therapy by Response Evalutation Criteria in Solid Tumors (RECIST) or Positron Emission Tomography Response Criteria in Solid Tumors (PERCIST) criteria (25, 26) and having a PDX model attempted in immune-deficient mice directly from patient biopsy. At the outset, selection was limited to patients with successful PDX modeling to streamline data review (Supplementary Fig. S1). This was later extended to include three patients with evaluable clinical response but no matched PDX model. Sex was not accounted for in this study, and no sex-based analyses were performed

### Patient response to treatment

Patient response was evaluated as previously described (1). Objective responses (OR) were evaluated by local board-accredited radiologists at participating centers and central review using the revised RECIST (25) or PERCIST (26) guidelines. Briefly, for RECIST, CR refers to disappearance of all lesions, PR refers to >30% decrease in the sum of the diameters of target lesions, taking as reference the baseline sum diameters, PD refers to >20% increase in the sum of the diameters of target lesions, taking as reference the smallest sum on study or the appearance of one or more new lesions, and SD is neither sufficient shrinkage to qualify for PR nor sufficient increase to qualify for PD, taking as reference the smallest sum diameters. For PERCIST, CR refers to 2-[18F]fluoro-2-deoxy-D-glucose uptake indistinguishable from surrounding background and less than liver, PR refers to a decrease of standardized uptake value (SUV) by >30%, SD refers to an increase or decrease of SUV by <30%, and PD refers to an increase of SUV by >30% or development of new lesion. For SD, measurements must have met the SD criteria at a minimum interval of 6 weeks after commencing a recommended treatment. Patients with a CR or a PR had confirmation of the response with a follow-up scan.

### Mouse xenograft studies

All mouse studies were performed in accordance with the guidelines approved by the University of New South Wales Animal Care and Ethics Committee (ACEC 19/82B, 20/119B) and the requirements of the Australian Code for the Care and Use of Animals for Scientific Purposes. Animals were housed in a specific pathogen-free environment, with two to six mice/cage and under 12:12 light/dark cycles with environmental enrichment. Irradiated rat and mouse breeder cubes and water were provided ad libitum. All animal procedures were performed inside a biosafety cabinet.

PDX models for extracranial tumors were established by subcutaneous implantation of a small tumor piece dipped in growth factor–reduced Matrigel into the dorsal flank of female NOD.Cg-Prkdc^scid^ Il2rg^tm1Wjl^/SzJ (NSG) mice (6, 8). Tumor growth was measured by Vernier caliper at least twice a week. Mice were euthanized once tumor size reached 1,000 mm^3^ (defined as engrafted), and tumor pieces were engrafted in secondary recipient mice for drug efficacy testing. Failure to engraft was defined as no measurable tumor 12 months after implantation (maximum holding time) and was confirmed by necropsy.

Drug treatment in secondary recipients commenced when a tumor reached a volume of 100 mm^3^, and event was defined as a quadrupling of tumor volume from the start of treatment. The OR was evaluated as previously described (27). Briefly, maintained complete response was defined as tumor volume <0.10 cm^3^ for 3 weeks from the cessation of treatment; CR was defined as disappearance of measurable tumor mass (<0.10 cm^3^) for at least one time point; PR was defined as regression ≥50% for at least one time point but with measurable tumor (≥0.10 cm^3^); SD was defined as <50% regression from initial volume during the study period and ≤25% increase in initial volume at the end of the study; and PD was defined as <50% tumor volume regression from initial volume during the study period and >25% increase in initial volume at the end of study period. The overall OR of each treatment was based on the median response of the group.

### *In vitro* high-throughput drug screening

Screening was conducted as previously detailed (6, 10). Briefly, dissociated cells from patient samples or cognate PDXs or *in vivo* expanded cells were seeded in 384-well assay plates as single-cell suspensions at 2,000 cells/well (12,500 cells/well for RA-049). After 3, 24, or 72 hours of incubation, the cells were treated with drugs approved or in preclinical development for pediatric cancer. Drugs were tested in duplicate in five different concentrations (10-fold serial dilutions; 0.5–5,000 nmol/L). After 72 hours of drug exposure, cell viability was measured using the CellTiter-Glo Luminescent cell viability assay (Promega) according to the manufacturer’s instructions. IC_50_ concentrations and area under the dose–response curve (AUC) values were calculated using the curve fitting parameters, with IC_50_ being defined as the drug concentration resulting in 50% cell survival. Sensitivity results were compared against an in-house reference database of responses using *z*-score methodology, in which the mean and variance of each compound in the database were compared against the corresponding AUC and IC_50_ values of the patients. A drug was considered a hit if the *z*-score of the AUC and IC_50_ was ≤ −2 (two standard deviations lower than the cohort mean). The cells used in screening were authenticated and validated by comparison with the donor tumor using short tandem repeat/microsatellite DNA profiling and copy number variation profile (Illumina Infinium Global Screening Array-24 v2.0).

### Zebrafish husbandry

Adult *casper* (28) zebrafish were maintained in a recirculating commercial housing system (Aquatic Habitats, now Pentair) at 28°C in 14 hour:10 hour light:dark conditions in the aquatics facility at the University of Ottawa, Ottawa, Canada. Adult *casper* zebrafish were bred according to standard protocol (29), and embryos were collected and grown in E3 medium (5 mmol/L NaCl, 0.17 mmol/L KCl, 0.33 mmol/L CaCl_2_, and 0.33 mmol/L MgSO_4_) at 28°C in 10-cm Petri dishes until the desired time point. Embryos were cleaned and provided with new media every 24 hours. The use of zebrafish in this study was approved and carried out according to the policies of the University of Ottawa’s Animal Care Committee (protocol #CHEOe-4241), which is governed and certified by the Canadian Council on Animal Care.

### Zebrafish larvae toxicity assay

All drugs used in this study were first dissolved in their appropriate solvents, with dimethyl sulfoxide being used for most drugs, expect for cyclophosphamide (cyclo), which was dissolved in saline. For treatment, the drug stock solutions were added to the embryo medium for immersion therapy in the larval zebrafish. The toxicity of each drug of interest was assessed *in vivo* on *casper* zebrafish larvae through immersion therapy. *Casper* zebrafish larvae at 72 hours post-fertilization (hpf) were arrayed one larva per well in a 96-well plate and treated once with increasing concentrations of each single agent of interest and the corresponding solvent control for 72 hours at 35°C. Larvae (*n* = 16) were tested per concentration or solvent control. For secondary verification of experimental doses, single and combination agents were confirmed in medium sized plates (60 × 15 mm) with 30 larvae per plate. The treating dose (Supplementary Table S1) was determined as 80% of the maximum tolerated dose (MTD) after 3 days of treatment. MTD is defined as the highest dose not causing general toxicity or death.

### Tumor cell preparation for larval zebrafish xenotransplantation

Samples were received as either cryopreserved tissue or cryopreserved single cells. Cryopreserved tissue samples were thawed and minced into small pieces and then transferred to a gentleMACS C-Tube (Miltenyi Biotec, #130-093-237) for dissociation using a tumor dissociation kit (Miltenyi Biotec, #130-095-929) and a gentleMACS dissociator (Miltenyi Biotec, #130-093-235). Dissociated single cells were processed through a dead cell removal kit (Miltenyi Biotec, #130-090-101) to eliminate cell debris, dead cells, and dying cells. Cryopreserved single-cell samples were thawed and processed through a dead cell removal kit. Viable single cells were labeled with CellTracker Deep Red cytoplasmic fluorescent dye (Thermo Fisher Scientific, #C34565) according to the manufacturer’s instructions. The cells were resuspended in DMEM supplemented with 10% heat-inactivated FBS for injection.

*Casper* zebrafish larvae at 48 hpf were anesthetized with 0.3mg/mL tricaine (MilliporeSigma, #A5040) and arrayed in troughs of an agarose injection plate for cell transplantation using protocols described previously (18, 30). The cells were backloaded into a pulled capillary needle and allowed to settle for approximately 20 minutes at 35°C to ensure a cell pellet at the bottom of the needle. A PLI-100A Picoliter Microinjector (Warner Instruments) was used to manually inject 100 to 200 cells into the yolk sac (YS) of each larva. Following injections, the larvae were kept at 35°C for the remainder of the experiment.

### Treatment and drug response evaluation in the larval zebrafish PDX

One day post-injection (dpi), larvae were screened on the Axio Zoom.V16 microscope (Carl Zeiss) under a far-red filter (620–700 nm) to ensure the presence of human tumor cells only present in the YS. To minimize bias, injected fish that passed screening were randomly and blindly distributed into treatment groups of 30 to 35 for treatments with either solvent control, single agent, or drug combinations by immersion therapy for 72 hours.

For drug response evaluation, *ex vivo* tumor cell quantification was performed at 1 dpi (baseline/untreated larvae) and 4 dpi (endpoint). Approximately 12 to 20 larvae from each treatment group were euthanized by an overdose of tricaine anesthetic. Whole larvae were dissociated in 100 mg/mL collagenase (Sigma-Aldrich) solution for 20 to 30 minutes in a heat block at 37°C. FBS (200 µL) was then added to slow the enzymatic reaction. The suspension was then centrifuged for 5 minutes at 300 × *g*. The supernatant was removed and the pellet containing fluorescent human tumor cells and zebrafish cells was washed with 30% FBS/PBS. The cells were then resuspended in 10 µL per embryo of 30% FBS/PBS solution for imaging. Then 10 µL boli were pipetted onto a 12-well printed slide (VWR, #100488-906) and imaged under a far-red filter using a 3 × 3 mosaic. The samples were imaged using an inverted Axio Observer microscope (Carl Zeiss) and images were analyzed using FIJI software (RRID: SCR_002285) to determine fluorescent cell number per group. The OR was evaluated based on a threshold created from the SEM of 1 day post-injection control (initial number of cancer cells). The overall OR of each treatment was based on the mean ± SEM response of the group; if error bars of treatment groups overlap with the threshold, they are not statistically different from 1 dpi. Briefly, PD refers to treatment groups with mean over the threshold (increase in the number of cancer cells); response refers to treatment groups with mean below the threshold (inhibition of cancer cells)—this is equivalent to CR and PR of patient clinical response; and SD refers to treatment groups with mean that falls within the threshold (mean number of cancer cells equivalent to the initial mean number of cells).

To confirm viability of cells in the larval zebrafish PDX, control 4 dpi were fixed in 4% paraformaldehyde overnight in 4°C. The samples were then embedded and oriented in 1% low melting point agarose and processed and embedded in paraffin and then sectioned to five-micron-thick sections using Leica RM2255. The obtained sections were deparaffinized using xylene and rehydrated in a gradient of ethanol. Endogenous peroxidase was blocked using 3% hydrogen peroxide. The slides were then stained using the standard procedure with 4′,6-diamidino-2-phenylindole (DAPI). Slides were mounted in fluoromount aqueous mounting medium (Sigma) and imaged using an inverted Axio Observer microscope (Carl Zeiss).

### Statistical analysis

Analyses for all studies were performed using GraphPad PRISM 9.0 and 10.2 software (RRID: SCR_005375). Larval zebrafish PDX datasets were analyzed using a one-way ANOVA with the Dunnett multiple comparison test comparing the mean of each group with the 4 days post-injection control group to identify the significance of treatment group responses. Differences were considered significant when *P* value was < 0.05 and statistical output was represented as follows: non-significant (ns) ≥ 0.05; *, < 0.05; **; < 0.01; ***, < 0.001; ****, < 0.0001. Mouse PDX survival curves were estimated for each treatment using the Kaplan–Meier method and compared with the untreated control group in each PDX model statistically using the log-rank test. *P* value for the log-rank test for comparison on EFS was non-significant (ns) ≥ 0.05; *, < 0.05; **, < 0.01. Heatmap comparing the three models was generated using Python code (RRID: SCR_024202), specifically leveraging libraries such as Matplotlib (RRID: SCR_008624) for data visualization.

### Data availability

All raw data generated in this study are available upon request from the corresponding authors.

## Results

### Patient enrollment and establishment of preclinical models

Samples from 10 high-risk children, adolescent, and young adult patients with cancer diagnosed with various tumor types and enrolled on the ZERO program were selected for this study. Criteria for inclusion were an evaluable clinical response—CR, PR, or SD—or a lack of response [progressive disease (PD)] to a single-agent or combination therapy by RECIST or PERCIST criteria (25, 26) and having a PDX model attempted in immune-deficient mice directly from patient biopsy (Table 1). As part of the ZERO study (1–5), all samples underwent molecular profiling including whole genome sequencing and RNA sequencing analysis, from which molecular targets were identified. The cohort consisted of three Ewing sarcomas, two soft-tissue sarcomas, two neuroblastomas, one osteosarcoma, one gastrointestinal stromal tumor, and one anaplastic large cell lymphoma. The patient age range was 1.5 to 15 years. For seven of 10 samples, a PDX model was successfully established in NSG mice, with initial engraftment time (to 1,000 mm^3^ tumor) of 37 to 182 days. Engraftment was unsuccessful for the remaining three samples, with no evidence of disease at the maximum holding time of 12 months after the engraftment attempt. Single-agent high-throughput screening (HTS) with a 125-drug library was previously conducted on seven of 10 patient samples (Supplementary Fig. S2; ref. 10). With this knowledge in hand, we transplanted samples into larval zebrafish and directly compared tumor drug responses to those of the donor patients and their cognate PDX models.

**Table 1:** Patient cohort details, including molecular targets, clinical response to therapy, and mouse PDX development

ID	Age (years)	Sex	Diagnosis^a^	Molecular target^b^	Evaluable clinical response^e^	Mouse PDX time (days)^c^	Single-agent HTS
zccs505	15	M	ES (relapse)	EWSR1:FLI1 fusion	Cyclo/topo (CR) Cyclo/topo/cabozantinib (CR)	49	Yes
zccs250	14	M	ALCL (diagnosis)	NMP1–ALK fusion	Ceritinib (CR)	37	No
zccs51	13	F	NBL (diagnosis)	*ALK* ^F1245I^ mut	Lorlatinib (PR)	182	Yes
zccs59	12	F	ES (relapse)	ETV1:EWSR1 fusion PIK3CA mut	IRN/TMZ/temsirolimus (PR)	67	Yes
zccs373	1.8	M	NBL (relapse)	MYCN amp	Cyclo/topo/venetoclax (PD) IRN/TMZ/alisertib (PD) IRN/TMZ/anti-GD2 (PD) Arginase (BCT-100) (PD)	73	Yes
zccs262	15	M	SARC (relapse)	ATM mut	Pazopanib (PD)	38	Yes
zccs43	15	M	OS (diagnosis)	TSC2 mut TP53–LSMD1 fusion	IRN/TMZ/temsirolimus (PD)	87	Yes
zccs170	1.5	M	RMS (relapse)	High FGFR4, AKT2, and MAP2K2	Temsirolimus/vinorelbine/cyclo (PR)	MHT^d^	No
zccs15	13	M	GIST (relapse)	SDHB loss SOS1 mut	Venetoclax (SD) Regorafenib (SD)	MHT^d^	No
zccs276	11	M	ES (relapse)	STAG loss	Olaparib/TMZ (PR)	MHT^d^	Yes

a ALCL, anaplastic large cell lymphoma; amp, amplification; ES, Ewing sarcoma; F, female; GIST, gastrointestinal stroma tumor; M, male; mut, mutant; NBL, neuroblastoma; OS, osteosarcoma; RMS, rhabdomyosarcoma; SARC, sarcoma.

b From whole-genome sequencing and RNA sequencing.

c Primary passage in mice (to 1,000 mm^3^ tumor).

d MHT = no engraftment at maximum holding time (12 months).

e Cyclo, cyclophosphamide; IRN, irinotecan; Topo, topotecan; TMZ, temozolomide.

### Drug tolerability in larval zebrafish

Prior to establishment of larval zebrafish PDX models, 23 single drugs and 13 drug combinations used to treat the 10 patients and their cognate mouse PDX models were tested for tolerability in the larval zebrafish. Notably, even at this early developmental time point, larval zebrafish have functional livers, kidneys, and a blood–brain barrier (31), as well as drug-metabolizing enzymes and metabolic rates comparable with humans (32). All drugs were water soluble and were delivered to the zebrafish larvae through immersion. MTDs were determined for single-dose 3-day treatment as the highest dose not causing systemic toxicity or death. Combination MTD doses were similarly determined. The immersion (treating) doses were defined as 80% of the MTD for each drug. Although drug uptake is likely to vary between agents, the immersion doses used were within the range of the maximum plasma concentration identified in human studies (33) as the highest recommended single dose (Supplementary Table S1).

### Establishment of larval zebrafish PDX models

We pursued the establishment of larval zebrafish PDX models for each of the 10 patients in the cohort to determine technical feasibility and concordance with responses observed in patients and mouse PDX models. Cryopreserved tissue or single cells from the original patient sample, or following expansion in NSG mice in Australia, were shipped to Canada to generate zebrafish larvae PDX models. For two samples (zccs250 and zccs59), further expansion in NSG mice was conducted prior to larval zebrafish PDX generation. Tumor cells were processed to remove dead or apoptotic cells, labeled with CellTracker Deep Red for visualization and quantification, and then injected into the YS of the larval zebrafish. The YS, a commonly used site for larval xenograft injection, offers a nutrient-rich environment for tumor cells, allowing cell growth and migration. Despite variability in cell viability (10%–84%) and yield, potentially due to the age or cryopreservation of the samples, we were still able to successfully generate larval zebrafish PDX models for all 10 patients (Table 2), including the three samples that could not be established as mouse PDX models, highlighting the robustness of the approach and the low viable cell number requirements. Larval zebrafish PDX models were then treated for 3 days with the same single agent or drug combinations received by patients and their cognate mouse PDX models, followed by an *ex vivo* tumor cell quantification for drug response evaluation (Fig 1B).

**Table 2:** Larval zebrafish PDX development: sample source and cell viability

ID	Sample source	Sample type	Cell viability (%)^a^	Labeled live cells (×10^6^)^b^	Larval zebrafish PDX	
					Generated	Significant growth in cancer cell number
zccs505	Mouse PDX	Tissue	44.6	2.5	Yes	Yes
zccs250	Mouse PDX^c^	Cells	30	1.5	Yes	Yes
zccs51	Mouse PDX	Cells	48	2.4	Yes	No
zccs59	Mouse PDX^c^	Cells	84	4.2	Yes	Yes
zccs373	Mouse PDX	Cells	10	0.5	Yes	No
zccs262	Mouse PDX	Tissue	15	0.1	Yes	Yes
zccs43	Mouse PDX	Cells	56.6	3	Yes	Yes
zccs170	Patient tumor	Cells	15.4	0.7	Yes	No
zccs15	Patient tumor	Cells	32.3	1.3	Yes	Yes
zccs276	Patient tumor	Cells	55.5	3.2	Yes	No

a Cell viability percentage using a trypan blue stain.

b Number of labeled live cells prior to injections.

c Underwent secondary expansion in Canada prior to larval zebrafish PDX generation.

### Larval zebrafish PDX modeling in clinical responders with established mouse PDX models

Of the seven of 10 patients for whom a mouse PDX model was successfully established, four had an evaluable objective clinical response to therapy (zccs505, zccs250, zccs51, and zccs59). For each patient, mouse PDX drug efficacy testing was guided by molecular sequencing findings, single-agent HTS, and treatments received by each patient. Each of these treatment regimens was assessed in a larval zebrafish PDX model to allow comprehensive comparison of responses.

**Figure 2:**
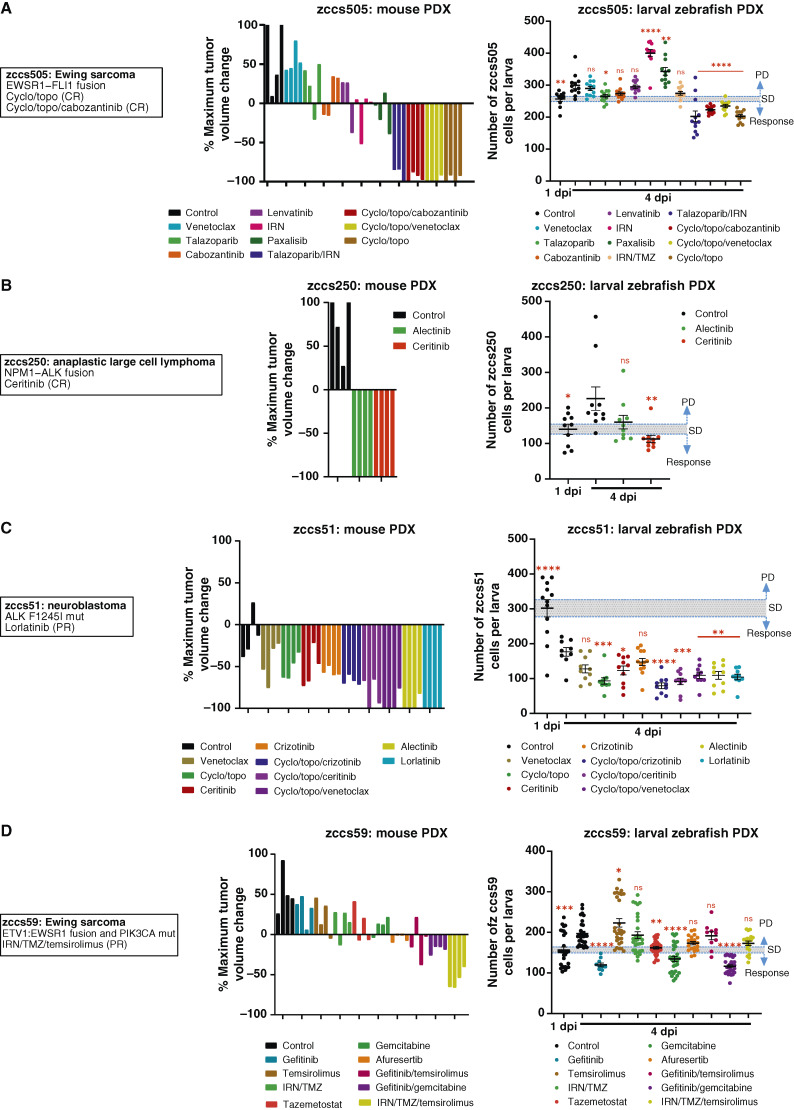
Mouse and larval zebrafish PDX drug efficacy for responsive patients. **A to D,** Patient information, alongside mouse PDX waterfall plot of maximum tumor regression, and larval zebrafish tumor cell numbers for each therapy for Ewing sarcoma zccs505 (**A**), anaplastic large cell lymphoma zccs250 (**B**), neuroblastoma zccs51 (**C**), and Ewing sarcoma zccs59 (**D**). For mouse PDX data, colored columns represent individual mice. For larval zebrafish data, error bars represent mean ± SEM, each colored dot represents an individual larva, and a threshold is based on SEM of 1 dpi. Statistical analysis was conducted using a one-way ANOVA with the Dunnett multiple comparisons test, comparing the mean of each treatment group with control (4 dpi), *P* values: *, < 0.05; **, < 0.01; ***, < 0.001; ****, < 0.0001. Treatments are ordered and colored the same in mouse and zebrafish studies. mut, mutant; dpi, days post-injection.

A patient with metastatic relapsed Ewing sarcoma, zccs505, with an *EWSR1–FLI1* gene fusion and previous disease progression following radiotherapy and irinotecan/temozolomide (IRN/TMZ) experienced CR following treatment with cyclophosphamide/topotecan (cyclo/topo) and cyclo/topo in combination with the multi-tyrosine kinase inhibitor cabozantinib, which was identified and informed by single-agent HTS (Supplementary Fig. S2). The cognate mouse PDX for zccs505 also experienced CR following cyclo/topo/cabozantinib and maintained complete response following cyclo/topo (Fig. 2A; Supplementary Fig. S3A). The cognate zccs505 zebrafish PDX model recapitulated this response following both of these combination treatment regimens, with a significant reduction in cell number compared with the initial number of cancer cells transplanted, in each case (Fig. 2A). Notably, although cabozantinib had been identified by single-agent HTS (Supplementary Fig. S2), cabozantinib alone resulted in PD in both mouse and zebrafish PDXs (Fig. 2A). More broadly, a high degree of concordance was observed between the mouse and zebrafish PDX models for zccs505 (nine of 10 comparisons, including progression for untreated or vehicle controls, progression for most single-agent therapies including paxalisib, and responses for combination therapies including talazoparib/IRN and combinations with cyclo/topo; Fig. 2A).

Similarly, for zccs250 (patient with anaplastic large cell lymphoma) with an *NPM–ALK* fusion and clinical CR to the ALK inhibitor, ceritinib, therapeutic response was recapitulated in the mouse PDX and a significant response was observed in the zebrafish PDX model (Fig 2B; Supplementary Fig. S3B). For a patient with neuroblastoma zccs51 harboring an *ALK*^F1245I^ mutation and clinical PR to the ALK inhibitor, lorlatinib, the cognate mouse and zebrafish PDX models both demonstrated response to lorlatinib (Fig. 2C; Supplementary Fig. S3C). For this sample, a reduction in cancer cell number was observed in the control group at 4 dpi in the zebrafish PDX; however, this was consistent with the reduction in cancer cell number in the mouse PDX and the protracted time (182 days) it took for this sample to engraft in the mouse (Table 1). Again, most treatment responses were also concordant between the mouse and zebrafish PDX models for this tumor (six of nine comparisons; Fig. 2C).

One patient, zccs59, with Ewing sarcoma (ETV1:EWSR1 fusion and PIK3CA mutation) did not exhibit consistent drug responses in the larval zebrafish PDX compared with those observed in the patient (Fig. 2D). Although the clinical PR to IRN/TMZ/temsirolimus was recapitulated in the mouse PDX (Fig. 2D; Supplementary Fig. S3D), this response was absent in the zebrafish PDX, resulting in lower overall therapeutic response concordance between zebrafish and mouse PDX models for this sample (four of nine comparisons; Fig 2D).

### Larval zebrafish PDX modeling in clinical non-responders with established mouse PDX models

**Figure 3:**
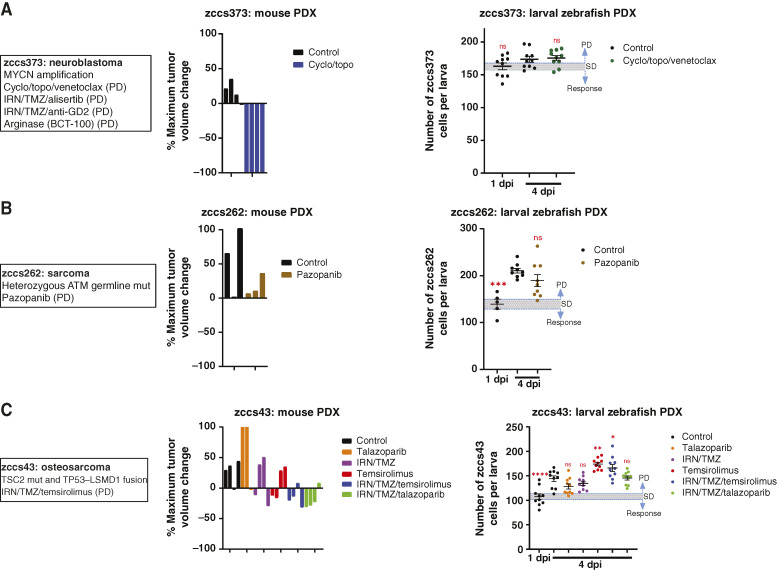
Mouse and larval zebrafish PDX drug efficacy for nonresponsive patients. **A to C,** Patient information, alongside mouse PDX waterfall plot of maximum tumor regression, and larval zebrafish tumor cell numbers for each therapy for neuroblastoma zccs373 (**A**), sarcoma zccs262 (**B**), and osteosarcoma zccs43 (**C**). For mouse PDX data, colored columns represent individual mice. For larval zebrafish data, error bars represent mean ± SEM, each colored dot represents an individual larva, and a threshold is based on SEM of 1 dpi. Statistical analysis was conducted using a one-way ANOVA with the Dunnett multiple comparisons test, comparing the mean of each treatment group with control (4 dpi), *P* values: *, < 0.05; **, < 0.01; ***, < 0.001; ****, < 0.0001). mut, mutant; dpi, days post-injection.

Of the seven of 10 patients for whom a mouse PDX model was successfully established, three patients (zccs373, zccs262, and zccs43) experienced evaluable clinical disease progression. A relapsed patient with neuroblastoma, zccs373 (*MYCN* amplified), was identified as potentially sensitive to the BCL2 inhibitor, venetoclax, and aurora kinase A inhibitor, alisertib, by single-agent HTS (Supplementary Fig. S2) but experienced disease progression following treatment with cyclo/topo/venetoclax. Consistent with the patient response, the larval zebrafish PDX did not respond to this combination (Fig. 3A). Although the two-drug combination of cyclo/topo was not tested in the patient or larval zebrafish PDX, unexpectedly, a CR was observed in the mouse (Fig. 3A; Supplementary Fig. S4A). A patient with relapsed metastatic undifferentiated sarcoma, zccs262 (harboring a heterozygous *ATM* germline mutation), experienced disease progression after treatment with pazopanib. This absence of response was recapitulated in both the mouse and zebrafish PDX models (Fig. 3B; Supplementary Fig. S4B). A patient with metastatic osteosarcoma, zccs43 (*TSC2* mutation and *TP53–LSMD1* fusion), experienced PD after treatment with IRN/TMZ in combination with temsirolimus, a finding reproduced in both mouse and zebrafish PDX models (Fig. 3C; Supplementary Fig. S3C). Furthermore, additional single-agent or combination treatments based on molecular findings were found to be concordant across both mouse and zebrafish PDX models for patient zccs43 (five of five comparisons), with PD/no response for talazoparib, temsirolimus, IRN/TMZ, and IRN/TMZ/talazoparib (Fig. 3C; Supplementary Fig. S4C).

### Larval zebrafish PDX as an alternative for difficult-to-engraft samples

**Figure 4:**
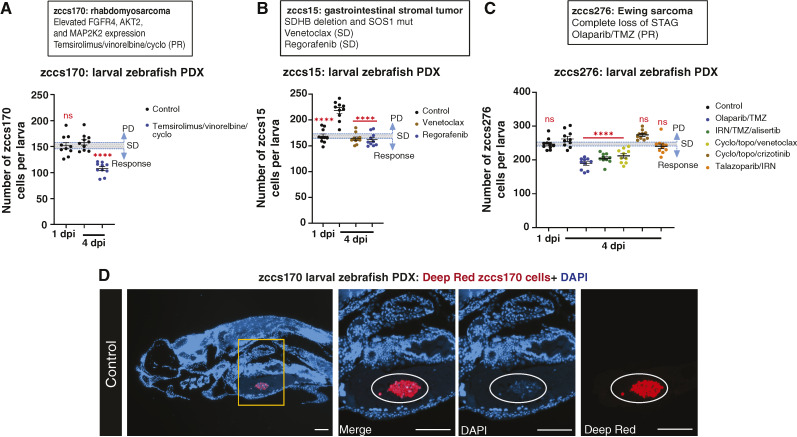
Larval zebrafish drug efficacy studies for patients with no available mouse PDX. **A to C,** Patient information, alongside larval zebrafish tumor cell numbers for each therapy for rhabdomyosarcoma zccs170 (**A**), gastrointestinal stromal tumor zccs15 (**B**), and Ewing sarcoma zccs276 (**C**). For larval zebrafish data, error bars represent mean ± SEM, each colored dot represents an individual larva, and a threshold is based on SEM of 1 dpi. Statistical analysis was conducted using a one-way ANOVA with the Dunnett multiple comparisons test, comparing the mean of each treatment group with control (4 dpi), *P* values: *, < 0.05; **, < 0.01; ***, < 0.001; ****, < 0.0001. **D,** Representative image of a five microns sectioned control larval zebrafish xenograft for patient zccs170. Deep Red–labeled patient cells present in the YS 4 dpi and labeled with DAPI to confirm viability. White circle in the images point to zccs170 tumor cells. Images were taken with 10× and 20× magnification. The scale bar is 100 µm. dpi, days post-injection.

For three of the 10 patients, zccs170, zccs15, and zccs276, a mouse PDX model could not be successfully established (Table 1), which precluded drug *in vivo* sensitivity testing for these patients. Despite the absence of a mouse PDX and despite not observing a significant increase in cancer cell numbers over 3 days under control conditions for two of the three samples in the larval zebrafish, robust drug sensitivity data were successfully collected. A patient with embryonal rhabdomyosarcoma, zccs170 (high *FGFR4*, *AKT2*, and *MAP2K2* mRNA expression), experienced a PR to temsirolimus/vinorelbine/cyclo. This response was consistent with the significant response to this combination observed in the zebrafish PDX model (Fig. 4A). Although there was minimal growth of zccs170 cancer cells in the zebrafish, 4′,6-diamidino-2-phenylindole (DAPI) staining of paraffin-embedded sections from these PDXs confirmed the viability of the patient cancer cells in the control group at 4 dpi (Fig. 4D). A patient with metastatic gastrointestinal stromal tumor, zccs15 (heterozygous germline *SDHB* deletion and *SOS1* mutation), experienced SD after treatment with single-agent venetoclax or regorafenib. In the zebrafish PDX model, both treatments individually prevented tumor cell expansion, analogous to disease stabilization (Fig. 4B). A patient with relapsed Ewing sarcoma, zccs276, with *STAG2* loss, which in a recent study that included both Ewing sarcoma mouse and larval zebrafish xenografts was associated with enhanced metastatic potential of Ewing sarcoma (34), experienced a PR of the target lesion to olaparib/TMZ with concurrent development of a new lesion before stopping treatment due to TMZ toxicity. The zebrafish PDX confirmed significant drug sensitivity to this combination treatment (Fig. 4C). Additionally, the patient sample showed sensitivity in single-agent HTS to talazoparib (PARP inhibitor), alisertib (AURKA inhibitor), crizotinib (ALK/ROS1/MET inhibitor), and venetoclax (Supplementary Fig. S2). These single agents in combination with conventional chemotherapy agents were tested for efficacy in the zebrafish PDX model, with significant responses to IRN/TMZ/alisertib and cyclo/topo/venetoclax; SD to talazoparib/IRN; and progression following cyclo/topo/crizotinib (Fig. 4C).

### Larval zebrafish PDX models effectively recapitulate drug responses observed in patients and mirror findings in mouse PDX models

**Figure 5:**
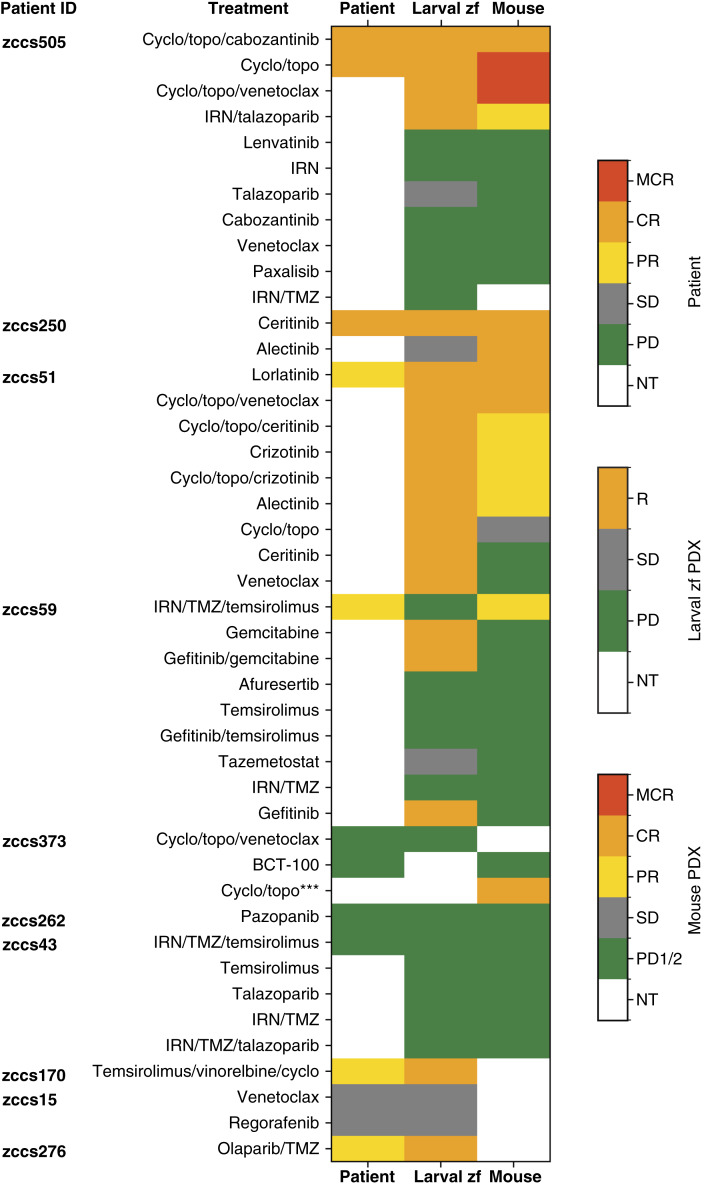
Consistency in drug response between patients and their cognate larval zebrafish and mouse PDX models. Evaluable response to therapy was compared for each patient and their cognate larval zebrafish PDX and mouse PDX models. Patient and mouse responses are indicated as OR criteria. Larval zebrafish PDX models are indicated as response (inhibition of cell growth), SD (cell number maintained), or PD (cell growth in the presence of drug). Maintained CR, CR, and PR for patient and mouse are considered equivalent to R (response) in the larval zebrafish model. The concordance of response for each model type with patient response is summarized in the legends on the right. ***For zccs373 (nonresponder to triple combination), mouse PDX responded to dual combination. MCR, maintained complete response; NT, not treated; zf, zebrafish.

Overall, a high degree of concordance was observed between evaluable patient responses and those observed in preclinical models whether established in larval zebrafish (92% concordance across 11 of 12 treatments; Fig. 5) or mouse (89% concordance across eight of nine treatments; Fig. 5). Notably, the concordance between zebrafish and mouse PDX models was significant, with 70% agreement across 26 of 37 treatments (Fig. 5).

## Discussion

This successful and unique international collaboration between the ZERO and PROFYLE studies has proven instrumental in demonstrating the predictive utility of the larval zebrafish platform to inform personalized treatment strategies for high-risk childhood cancers. Often in these cases, established treatment paradigms are not available, and tumor-directed therapy needs to be initiated quickly owing to the aggressive nature of these refractory and relapsed cancers, only for 70% of which can actionable molecularly guided treatments be identified (5, 6). Individualized patient models, or avatars, can help guide and prioritize management strategies not only for patients lacking actionable molecular aberrations but can also orthogonally provide functional validation of personalized treatment recommendations based on molecular profiling. Moreover, a lack of response may reveal the clinical futility of pursuing a particular treatment paradigm in an individual patient (6). Although the mouse PDX model is often considered the gold standard for preclinical testing, its often lengthy time to engraftment and failure to reliably engraft all tumor samples highlight its limitations in consistently providing real-time data to inform patient treatment. Here, we present compelling evidence that the larval zebrafish PDX platform can dependably provide robust real-time response data that are highly reflective of clinical responses of individual patients to single-agent and combination therapy and are nearly identical to that observed in conventional mouse PDX models, thus permitting prioritization of therapeutic options in a far more rapid and cost-effective manner.

In directly comparing the complementary preclinical modalities used in this study, several practical aspects of each approach become apparent (Supplementary Table S2). Critically, the larval zebrafish model was successful even for patients for whom a mouse PDX model could not be generated; viable cell numbers required for larval zebrafish testing were orders of magnitude lower than required for testing in a mouse PDX; and our larval zebrafish workflow was less than 1 week from transplantation to completion, a comparable time frame but with a greater predictive response in an *in vivo* model than *in vitro* HTS (10). In contrast, the mouse PDX models in our study required up to 182 days for the establishment of the initial engrafted tumor, and modeling was not achievable for some patients. In practice, even for successful mouse xenografts, this time is frequently much longer as limited patient tumor material, particularly from a core needle biopsy, can necessitate initial PDX establishment, followed by secondary *in vivo* expansion prior to drug efficacy testing, further increasing the time required to generate preclinical response data (6).

The rapid experimental time frame of the zebrafish PDX offers a crucial advantage for young patients with high-risk cancers, for whom timely decisions about potentially life-saving treatments are essential. This approach allows for rapid functional assessment of drug and drug combination efficacy, offering real-time insights while minimizing the risk of substantial tumor evolution in patients that could alter drug responses. Immersion therapy is an efficient and high-throughput approach for drug testing in the larval zebrafish; however, non-soluble compounds can be administered effectively by injecting into the circulation of the larval fish (via the duct of Cuvier) or via oral gavage, broadening the range of testable agents.

A limitation of the larval zebrafish PDX model is its reduced ability to capture the full granularity of treatment responses. Response classification is based on threshold-defined changes in cancer cell number and does not distinguish between complete and PRs as done in the clinical setting or mouse PDX models. Thus, although the zebrafish model supports rapid prioritization of therapeutic options, its results are best interpreted as early indicators requiring validation in longer-term models. Furthermore, survival studies are not feasible in larval zebrafish as PDXs are typically not maintained beyond 6 dpf. Beyond this stage, human tumor cells are unlikely to survive because of the development of the zebrafish’s adaptive immune system, which becomes functional around 28 dpf.

Although we observed that most patient responses were also seen in their cognate PDX models, not every case was concordant. Notably, the clinical PR to IRN/TMZ/temsirolimus observed for patient zccs59 was not replicated in the larval zebrafish PDX model, but was observed in the mouse PDX model. Possible explanations for this discrepancy may include clonal selection or evolution during additional expansion in mice. Zccs59 was one of two samples that underwent a second round of expansion prior to larval zebrafish PDX generation (Table 2). Further studies will be required to definitively determine the underlying cause. Comparative molecular profiling of donor and engrafted material should be incorporated into future studies to understand such divergences.

Importantly, we also noted a consistent relationship between mouse PDX engraftment time (Table 1) and growth kinetics in the larval zebrafish model (Table 2). Of the three samples that failed to engraft in mice, two (zccs170 and zccs276) demonstrated a limited increase in cell numbers in zebrafish, whereas the slowest mouse PDX model, zccs51, showed reduced cell count or cell death in both control zebrafish after 4 days and in control mouse PDXs during the drug efficacy experiments. Conversely and consistently, the most rapidly established mouse PDX models (zccs250 and zccs262) showed the greatest growth in cancer cell numbers in zebrafish.

This study represents the first proof-of-principle pediatric precision oncology study to directly compare drug efficacy testing in both mouse and larval zebrafish PDX models with patient clinical drug response data. Although the larval zebrafish work in this study was conducted retrospectively, the highly consistent and conserved responses observed in larval zebrafish PDX models make a strong argument for incorporating this platform up front in therapeutic pipelines. The larval zebrafish model holds a significant promise for expediting preclinical testing and improving the efficiency of evaluating personalized treatment options for the greatest number of pediatric patients with cancer, providing much-needed hope and enhancing the quality of life for these most vulnerable patients.

## Supplementary Material

Figure S1 CONSORT flow diagram for patients included in the study and the criteria not met for those excluded

Table S1 Single agent and combination doses established in the larval zebrafish PDX at 35°C for 3 to 7 days post fertilization zebrafish for 72-hour treatment.

Figure S2 Ex vivo single agent high-throughput screening of a 125-drug library in patient-derived samples.

Figure S3 Mouse PDX tumor growth curves and Kaplan-Meier survival curves

Figure S4 Mouse PDX tumor growth curves and Kaplan-Meier survival curves

Table S2 Features of mouse PDX, larval zebrafish PDX and in vitro models
