# Supplementary material for: Modeling High-Risk Pediatric Cancers in Zebrafish to Inform Precision Therapy
Source: Cancer Res Commun. 2025 Jul 25;5(7):1215–27. doi: 10.1158/2767-9764.CRC-25-0080 (PMC12290838; doi:10.1158/2767-9764.CRC-25-0080)
Supplement: Figure S1 — CONSORT flow diagram for patients included in the study and the criteria not met for those excluded [file crc-25-0080_figure_s1_suppsf1.pdf]

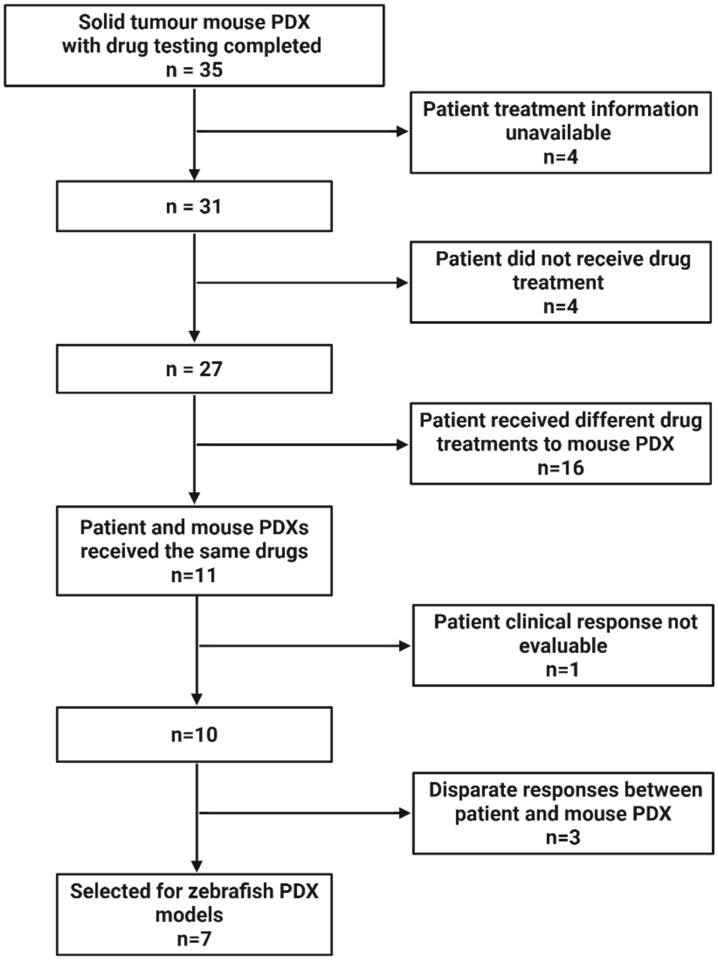

Fig. S1. CONSORT flow diagram for patients included in the study and the criteria not met for those excluded. At the outset, selection was limited to patients with successful PDX modeling to streamline data review. This was later extended to include three patients with evaluable clinical response but no matched PDX model, with a total of n=10 selected for zebrafish PDX models.
