# Supplementary material for: Modeling High-Risk Pediatric Cancers in Zebrafish to Inform Precision Therapy
Source: Cancer Res Commun. 2025 Jul 25;5(7):1215–27. doi: 10.1158/2767-9764.CRC-25-0080 (PMC12290838; doi:10.1158/2767-9764.CRC-25-0080)
Supplement: Table S1 — Single agent and combination doses established in the larval zebrafish PDX at 35°C for 3 to 7 days post fertilization zebrafish for 72-hour treatment. [file crc-25-0080_table_s1_suppst1.pdf]

Table S1. Single agent and combination doses established in the larval zebrafish PDX at 35°C for 3-7 days post fertilization zebrafish for 72-hour treatment.

Drug	Target/class	Larval zf treating dose	C_{max}[a]
Ceritinib	ALK, IGF1R	2 µM	1.21 µM
Alectinib	ALK	10 µM	1.38 µM
Lorlatinib	ALK	3 µM	N/A
Venetoclax	Bcl-2	5 µM	4.48 µM
Crizotinib	ALK, MET, ROS1	20 µM	0.913 µM
Topo	Topoisomerase I	10 µM	0.015 µM
Gemcitabine	Antimetabolite	70 µM	89.3 µM
Gefitinib	EGFR	10 µM	0.356 µM
Tazemetostat	EZH2	8 µM	N/A
Afuresertib	PI3K/AKT/mTOR	10 µM	N/A
Cabozantinib	Multi-TKI	75 nM	4.61 µM
Talazoparib	PARP	40 µM	N/A
Lenvatinib	Multi-TKI	50 nM	0.761 µM
Paxalisib	PI3K/AKT/mTOR	0.25 µM	N/A
Pazopanib	Multi-TKI	0.25 µM	133 µM
Alisertib	AKA	10 µM	N/A
Regorafenib	Multi-TKI	0.08 µM	8.08 µM
IRN	Topoisomerase I	20 µM	5.78 µM
Temsirolimus	mTOR	0.25 µM	0.568 µM
TMZ	Alkylating agent	70 µM	37.6 µM
Cyclo	Alkylating agent	0.5mM	128 µM
Olaparib	PARP	40 µM	13.1 µM
Vinorelbine	Vinca alkaloids	50 µM	0.811 µM
**Drug combination**		**Larval ZF treating dose**	
Cyclo/Topo		0.5mM + 10 µM	
Cyclo/Topo/Crizotinib		0.5mM + 10 µM + 20 µM	
Cyclo/Topo/Ceritinib		0.5mM + 10 µM + 2 µM	
Cyclo/Topo/Venetoclax		0.5mM + 10 µM + 5 µM	
Cyclo/Topo/Cabozantinib		0.5 mM + 10 µM + 75 nM	
IRN/TMZ		20 µM + 70 µM	
IRN/TMZ/Temsirolimus		10 µM + 35 µM + 0.125 µM	
IRN/TMZ/Alisertib		10 µM + 70 µM + 10 µM	
IRN/TMZ/Talazoparib		10 µM + 35 µM + 40 µM	
Gefitinib/Gemcitabine		2 µM + 15 µM	
Gefitinib/Temsirolimus		5 µM + 0.25 µM	
Talazoparib/IRN		20 µM + 10 µM	
Temsirolimus/Vinorelbine/Cyclophosphamide		0.25 µM + 50 µM + 0.5 mM	
Olaparib/TMZ		40 µM + 70 µM	

[a]C_{max}, maximum plasma concentration in patients (*29*). ZF, zebrafish; AKA, Aurora A kinase; PI3K, phosphoinositide 3-kinase; ALK, anaplastic lymphoma kinase; mTOR, mammalian target of rapamycin; IGF1R, insulin like growth factor 1 receptor; Bcl-2, B-cell lymphoma 2; EZH2, enhancer of zeste homolog 2; TKI, tyrosine kinase inhibitor. Cyclo, Cyclophosphamide; Topo, Topotecan; IRN, Irinotecan; TMZ, Temozolomide.
