# Supplementary material for: Modeling High-Risk Pediatric Cancers in Zebrafish to Inform Precision Therapy
Source: Cancer Res Commun. 2025 Jul 25;5(7):1215–27. doi: 10.1158/2767-9764.CRC-25-0080 (PMC12290838; doi:10.1158/2767-9764.CRC-25-0080)
Supplement: Figure S2 — Ex vivo single agent high-throughput screening of a 125-drug library in patient-derived samples. [file crc-25-0080_figure_s2_suppsf2.pdf]

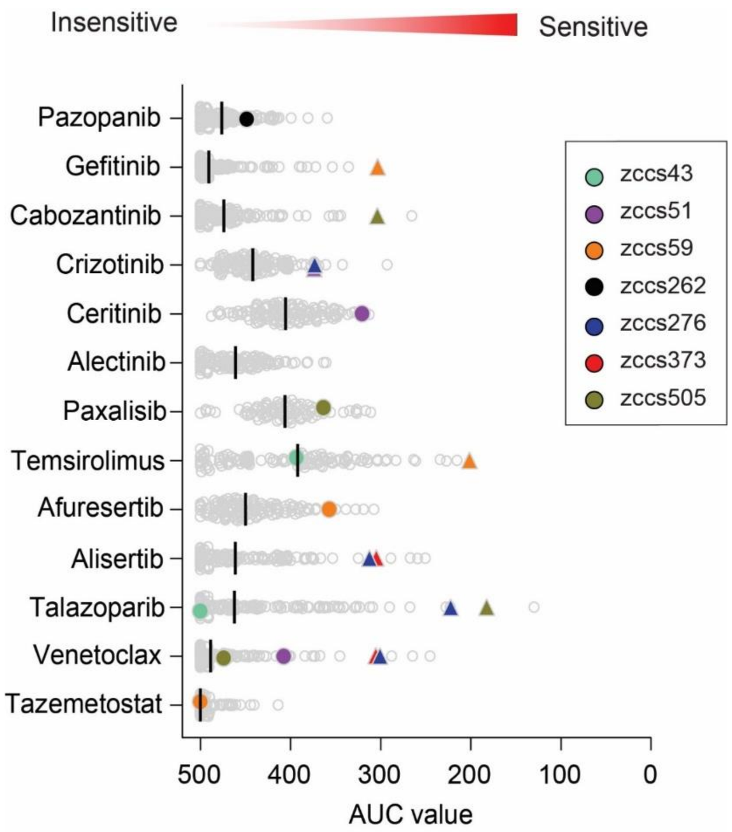

Fig. S2. *Ex vivo* single agent high-throughput screening of a 125-drug library in patient-derived samples. Dot plot presenting the AUC values of indicated targeted drugs across a 125-ZERO sample cohort, as detailed in Mayoh et al, 2023. The AUC values were calculated from dose-response curves established after 3 days of treatment. Dots are color coded to represent 7 distinct patient samples with matching available zebrafish PDX models. Triangle-shaped colored dots indicate reported drug hits for the 7 samples based on AUC and IC_{50} Z scores ≤ -2 and circular-shaped colored dots indicate drugs targeting a molecular alteration in the tumor or of (clinical) interest for a specific cancer type
