# Supplementary material for: Modeling High-Risk Pediatric Cancers in Zebrafish to Inform Precision Therapy
Source: Cancer Res Commun. 2025 Jul 25;5(7):1215–27. doi: 10.1158/2767-9764.CRC-25-0080 (PMC12290838; doi:10.1158/2767-9764.CRC-25-0080)
Supplement: Figure S3 — Mouse PDX tumor growth curves and Kaplan-Meier survival curves [file crc-25-0080_figure_s3_suppsf3.pdf]

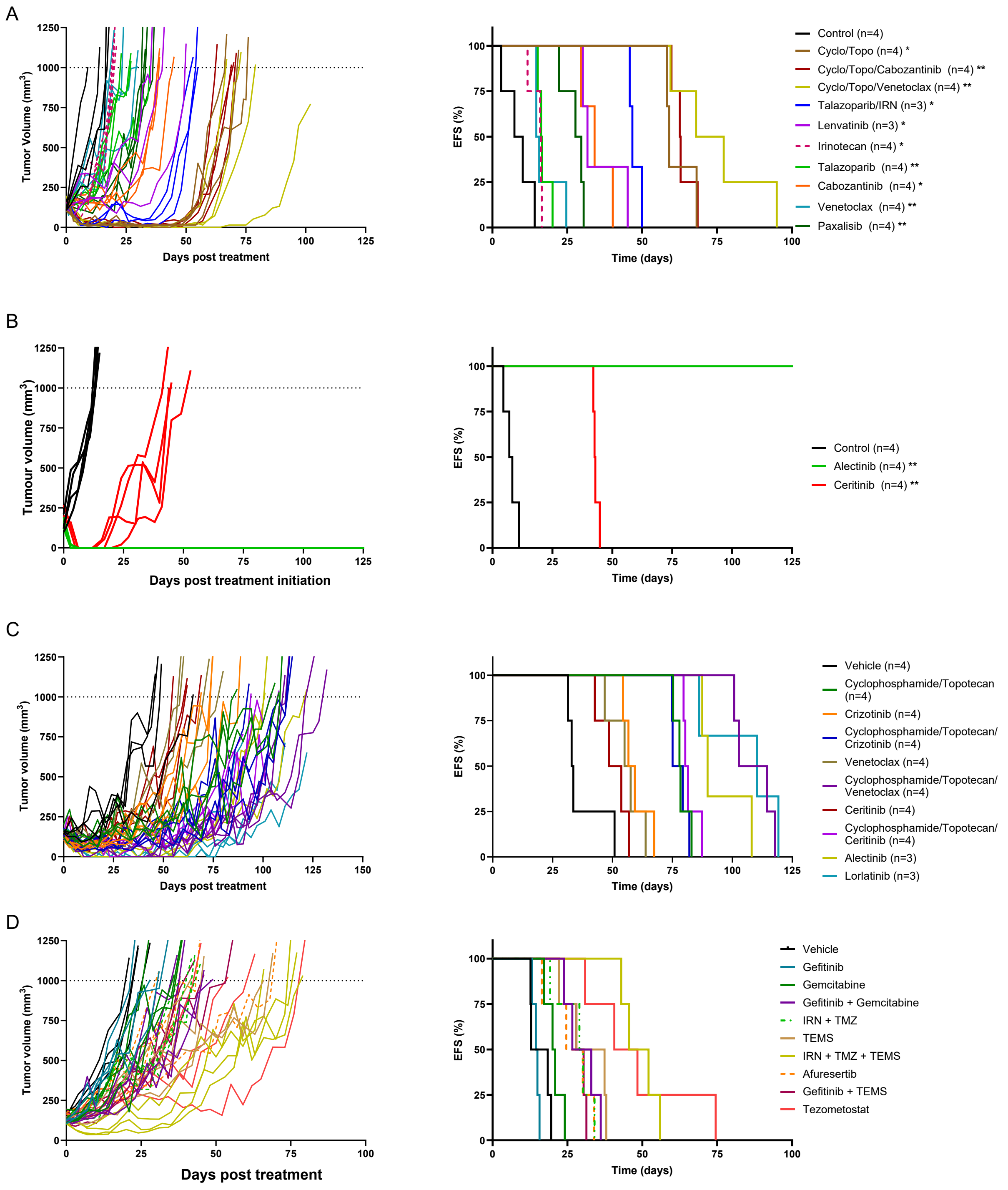

Fig. S3. Mouse PDX tumor growth curves and Kaplan-Meier survival curves for each therapy for Ewing sarcoma zccs505 **(A)**, anaplastic large cell lymphoma zccs250 **(B)**, neuroblastoma zccs51 **(C)**, and Ewing sarcoma zccs59 **(D)**. IRN, Irinotecan; TMZ, Temozolomide; TEMS, temsirolimus.
