# Supplementary material for: Modeling High-Risk Pediatric Cancers in Zebrafish to Inform Precision Therapy
Source: Cancer Res Commun. 2025 Jul 25;5(7):1215–27. doi: 10.1158/2767-9764.CRC-25-0080 (PMC12290838; doi:10.1158/2767-9764.CRC-25-0080)
Supplement: Figure S4 — Mouse PDX tumor growth curves and Kaplan-Meier survival curves [file crc-25-0080_figure_s4_suppsf4.pdf]

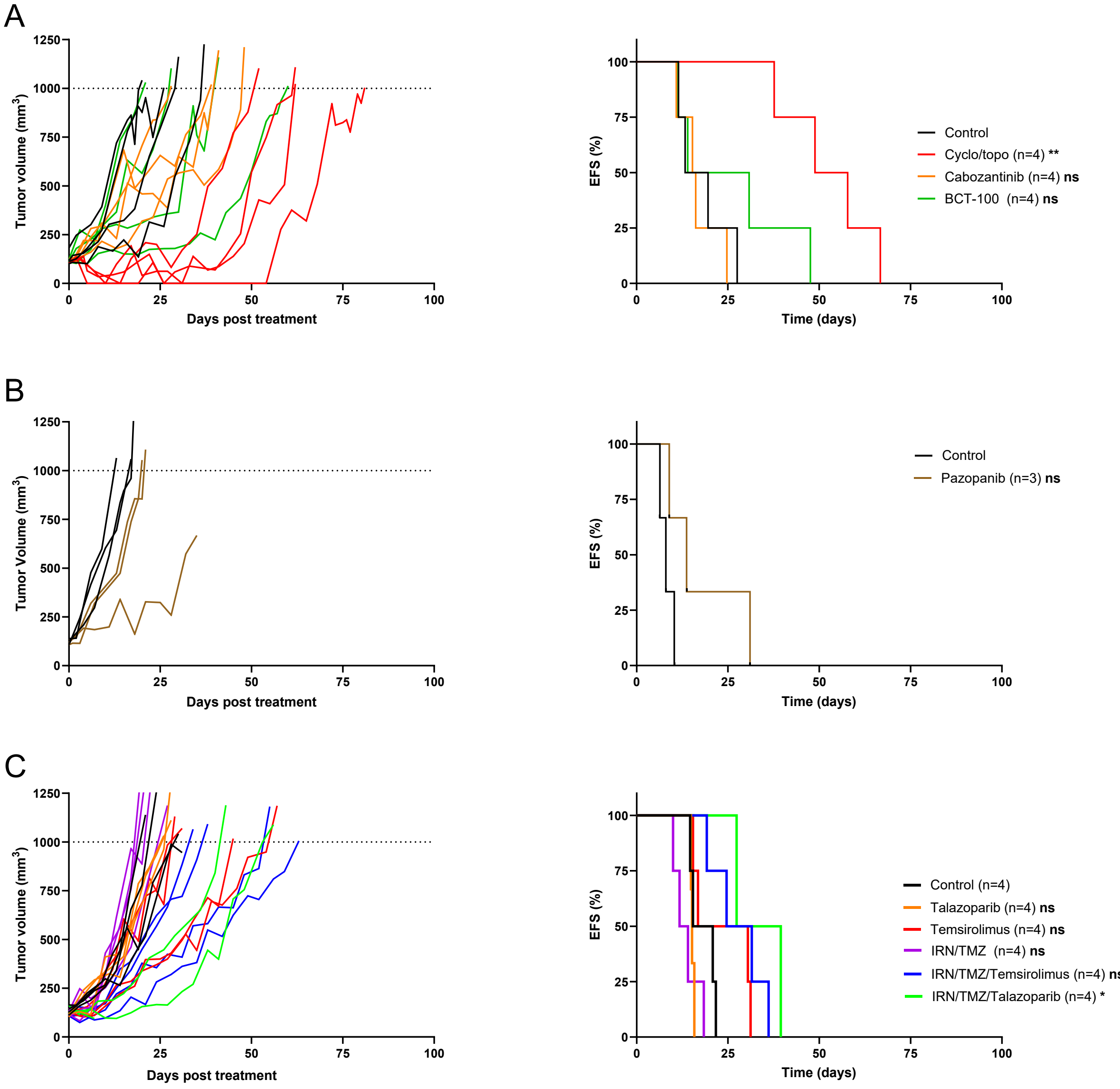

Fig. S4. Mouse PDX tumor growth curves and Kaplan-Meier survival curves for each therapy for neuroblastoma zccs373 **(A)**, sarcoma zccs262 **(B)**, and osteosarcoma zccs43 **(C)**. Cyclo, Cyclophosphamide; Topo, Topotecan; IRN, Irinotecan; TMZ, Temozolomide.
