# Supplementary material for: Modeling High-Risk Pediatric Cancers in Zebrafish to Inform Precision Therapy
Source: Cancer Res Commun. 2025 Jul 25;5(7):1215–27. doi: 10.1158/2767-9764.CRC-25-0080 (PMC12290838; doi:10.1158/2767-9764.CRC-25-0080)
Supplement: Table S2 — Features of mouse PDX, larval zebrafish PDX and in vitro models [file crc-25-0080_table_s2_suppst2.pdf]

Table S2. Features of mouse PDX, larval zebrafish PDX and *in vitro* models

	**Mouse** 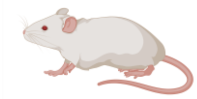	**Larval zebrafish** 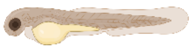	***In vitro*** 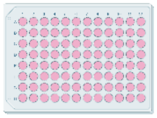
**Growth temperature**	37°C	35°C	37°C
**Throughput**	low	medium	high
**Animals/per group**	2–5	35–40	N/A
**Cells/animal**	1–5×10^6 cells	100–200 cells	N/A
**Experiment time**	months	days	days
**Cost**	$$$	$	$$
